# Characterization of muscle alteration in oral submucous fibrosis-seeking new evidence

**DOI:** 10.4317/medoral.20656

**Published:** 2015-10-09

**Authors:** Himanshi Chawla, Aadithya-Basavaraj Urs, Jeyaseelan Augustine, Priya Kumar

**Affiliations:** 1 MDS. Post Graduate student, Department of Oral & Maxillofacial Pathology , Maulana Azad Institute of Dental Sciences , New Delhi, India; 2 MDS, Professor and Head , Department of Oral & Maxillofacial Pathology, Maulana Azad Institute of Dental Sciences , New Delhi, India; 3 MDS, Associate Professor, Department of Oral & Maxillofacial Pathology, Maulana Azad Institute of Dental Sciences , New Delhi, India; 4 MDS, Associate Professor, Department of Oral & Maxillofacial Pathology, Maulana Azad Institute of Dental Sciences , New Delhi, India

## Abstract

**Background:**

The aim of the study was to assess the progression of Oral Submucous Fibrosis (OSF) by investigating the correlation between clinical mouth opening and muscle-epithelial distance in tissue sections. Characterization of changes involving muscle was ascertained.

**Material and Methods:**

50 cases and 10 controls were included in this case-control study. Inter-incisal mouth opening was measured and classified according to Lai *et al*. as Group A (more than 35mm), Group B (30 to 35mm), Group C (20 to 30mm), Group D (less than 20mm). Histopathological sections were graded as very early, early, moderately advanced, advanced OSF. Muscle-epithelial distance was calculated using image analysis software. The four most common degenerative changes observed in muscles, namely fragmentation, highly eosinophilic areas with loss of striations, nucleus internalization and multiple pyknotic nuclei were also assessed.

**Results:**

Comparisons of muscle-epithelial distance were made between the clinical and histopathological groups to those of controls. The mean muscle-epithelial distance was: Group A-626.8±309.36 µm, B-827.5±549.72 µm, C-673.2±321.93 µm, D-439.9±173.84µm, Controls-1222.19 ±441.7µm. Post-hoc Bonferroni Test revealed a statistically significant reduction in the muscle-epithelial distance in Group C (*p*-value = 0.001) and D (*p*-value = 0.001) as compared to controls. The mean muscle-epithelial distance in very early, early, moderately advanced and advanced OSF was 732.73±232.81µm, 726.54±361.63 µm, 548.36±273.13 and 172.40±58.41 µm respectively. Highly significant difference in muscle-epithelial distance was seen between controls as compared to early (*p*-value =0.002), moderately advanced (*p*-value = 0.001) and advanced OSF (*p*-value = 0.001. Fragmentation and highly eosinophilic areas were invariably noticed in advanced OSF. Multiple pyknotic nuclei were variable with no specificity.

**Conclusions:**

Reduction in muscle-epithelial distance may prove to be a significant predictor of OSF progression. Degenerative changes must be noted while observing OSF cases, irrespective of the histopathological grade.

**Key words:**Oral submucous fibrosis, muscle changes, muscle-epithelial distance.

## Introduction

Oral Sub mucous Fibrosis (OSF) is a potentially malignant disease predominantly seen in the people of South-Asian countries (1). The most common clinical symptoms associated with this disease are intolerance to spicy food, rigidity of lips, tongue and palate with limitation of mouth opening and tongue movements to varying degrees depending upon the severity of the disease (2). The histopathological changes associated with OSF have also been studied by many authors in the past. The characteristic histopathological features usually described with respect to OSF are excessive collagen deposition in the connective tissue, juxtaepithelial hyalinization, muscle degeneration and atrophic epithelium (3).

Few authors in the past have also given due reference to changes in the muscles observed in tissue sections of OSF. Binnie and Cawson (1972) noted that characteristic feature of OSF as seen under light microscope was a homogenous, collagenous sub-epithelial zone in which there were degeneration of muscle fibres (4). El-Lab ban and Canniff (1985) stated that restricted mouth opening in cases of OSF not only depends on sub epithelial fibrosis, but also on the extent of muscle damage and demonstrated various ultra structural muscle changes in patients with restricted mouth opening (5). Gupta *et al* (2000) found degenerative changes in the form of loss of cross striations, oedematous muscle fibres and atrophy in palatal and paratubal muscles in OSF patients (6). However, another distinctive finding noticed while carefully examining histopathological sections of OSF under light microscope is progressive decrease in the distance of muscle fibers from the epithelial surface as the lesion advances. There is a lack of evidence to support this common yet neglected finding. The present study was undertaken to discover a new parameter involving decrease in muscle-epithelial distance, which would correlate more effectively with clinical and histopathological grades, since usually the clinical grading does not match with the histopathological grading in OSF cases (7,8). Also, a detailed examination of various degenerative changes involving muscle fibres was undertaken. To the best of our knowledge, no researcher has previously examined changes in muscle to epithelium distance in OSF. This study is first of its kind which gives an elaborate detail of muscle changes in OSF.

The aim of the present study was to investigate correlation between clinical mouth opening and the distance of muscle from epithelium in tissue sections of patients with OSF. Correlation between muscle-epithelial distance and histopathological grade of OSF was also assessed. In addition, characterization of degenerative changes involving muscle was also ascertained.

## Material and Methods

The present study was undertaken at the Department of Oral Pathology, Maulana Azad Institute of Dental Sciences, New Delhi, India with prior approval of institutional ethical review committee. Informed consent was obtained from patients and controls participating in the study. A careful clinical examination of patients reporting to the department with signs and symptoms of OSF was performed with special concern to inter-incisal mouth opening. The distance between the incisal edges of maxillary right central incisor and mandibular right central incisor with patient’s mouth fully opened, was measured using vernier caliper. Three measurements were carried out in each case and the average value was calculated to give the final inter-incisal distance. The clinical staging scheme proposed by Lai *et al*. is as follows (1):

Group A: More than 35mm

Group B: Between 30 to 35 mm

Group C: Between 20 to 30 mm

Group D: Less than 20 mm

To make each interval exclusive for statistical analysis, the following clinical staging model was used:

Group A: ? 35 mm

Group B: Between 30 to 34 mm

Group C: Between 20 to 29 mm

Group D: ? 19mm

Age and sex matched healthy individuals were taken as controls. Incisional biopsy was then performed after taking patient’s (and controls’) consent. In both the study and the control groups, the biopsy site was chosen to be 1 cm behind the commissure of the mouth in order to remove any bias in the study due to difference in the depth of muscle at different locations in the buccal mucosa. All the tissues were fixed in 10% neutral buffered formalin for 12 hours prior to processing.

The sections were stained with hematoxylin and eosin and observed under microscope. All the tissues which were fragmented or did not show muscle fibres due to inadequate depth of the biopsy were excluded from the study. Exclusion criteria also included any associated dysplastic changes since hyperplasia may occur in such cases which may provide erroneous results while measuring distance. The samples were collected over a time period of six months. Stained sections in the study group were histopathologically graded as very early, early, moderately advanced and advanced OSF according to the criteria given by Pindborg *et al*. (9). The Motic image analysis software (Motic Images Plus 2.0) was used to calculate the distance of muscle from the rete ridge of the overlying epithelium. The shortest distance was measured from the crest of most superficial muscle fibre seen in a randomly chosen field to the bottom of deepest rete ridge. Three different histopathologists reviewed the slides simultaneously on image analysis software to remove any observer bias. The mean of distance in five such fields was referred as the muscle-epithelial distance for that particular case.

Wherever in doubt, Van Gieson stained sections were used to clearly delineate muscle fibres. Muscle-epithelial distance was also calculated in the control tissues for comparison.

While observing the histopathological sections, several degenerative changes in the muscle fibres were noted. Out of these, the four most commonly observed changes were:

a. Highly eosinophilic muscle fibres with loss of striations

b. Fragmentation

c. Nucleus internalization

d. Multiple nuclei

Each of the above mentioned observation was recorded as present or absent in every tissue section.

Statistical analysis was performed using Post-hoc Bonferroni Test to compare muscle-epithelial distance amongst various clinical groups as well as amongst different histopathological groups. One-way ANOVA test was used to compare clinical mouth opening and histopathological grade.

## Results

A total of 50 cases were included in the study with age ranging from 19 to 68 years. The condition affected males five times more commonly than females. The inter-incisal distance as measured clinically ranged from 8 mm to 40 mm. Group C [mouth opening 20-29 mm] comprised 24 patients followed by Group D [mouth opening 0-19 mm] with 19 patients. Group A [mouth opening 35 mm and above] and B [mouth opening 30-34 mm] had 3 and 4 patients each respectively.

The mean muscle-epithelial distance was ( Table 1): Group A-626.8 ± 309.36 µm, Group B-827.5 ± 549.72 µm, Group C-673.2 ± 321.93 µm, Group D-439.9 ± 173.84µm. The mean muscle epithelial distance in control group was calculated to be 1222.19 ± 19 µm. There was a statistically significant reduction in the muscle-epithelial distance in Group C [*P*-value = 0.001] and D [*P*-value = 0.001] as compared to control cases. Group A and B had a very small sample size each and therefore no significant results were obtained. However, when both the groups were merged; reduction in the muscle epithelial distance as compared to controls was statistically evident in this new group also [*P*-value = 0.016] ( Table 1). No significant results were obtained while performing intergroup comparison.

**Table 1:**
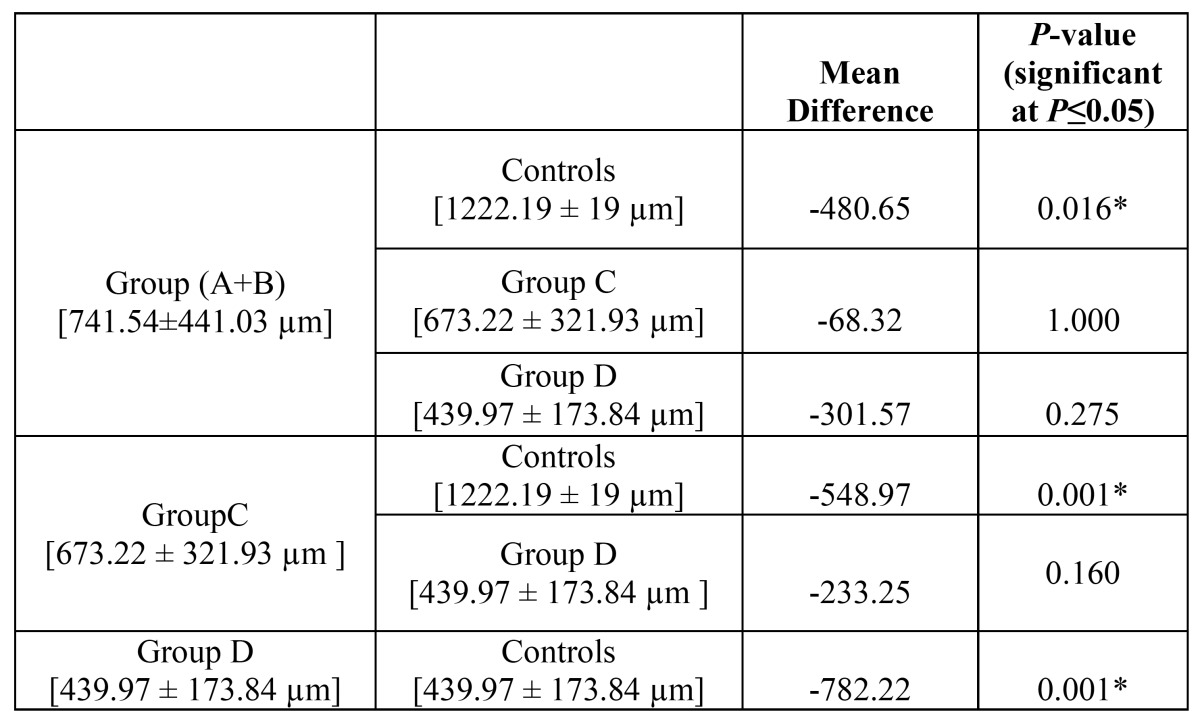
Comparison of muscle-epithelial distance amongst clinical groups using post-hoc Bonferroni Test.

**Figure 1:**
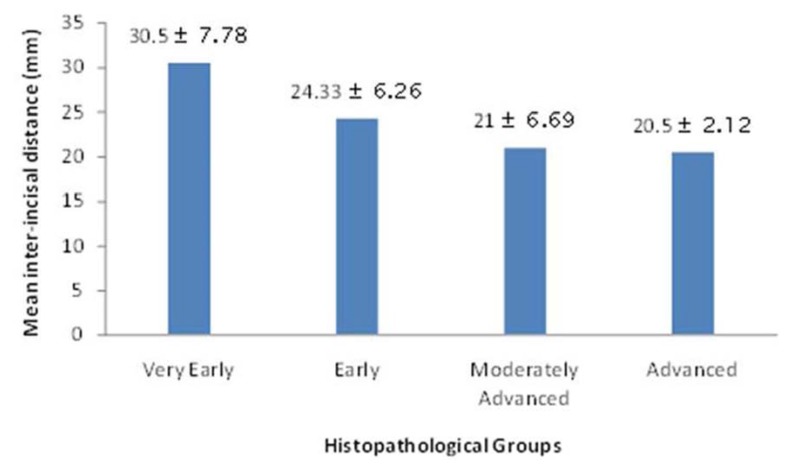
Graph showing comparison of mean inter-incisal distance in histopathological groups.

All the cases were also examined and classified histopathologically. Out of the 50 cases, maximum cases belonged to moderately advanced group [31], followed by early [15] and 2 cases each in very early and advanced group. The mean inter-incisal distance in each group was: 30.50 ± 7.78 mm in very early OSF, 24.33 ± 6.26 mm in early OSF, 21 ± 6.69 mm in moderately advanced and 20.50 ± 2.12 mm in advanced OSF (Fig. 1).

**Figure 2:**
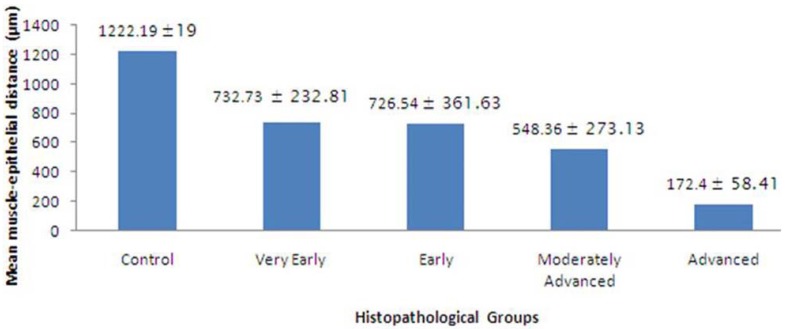
Graph showing comparison of mean muscle-epithelial distance in histopathological groups.

**Figure 3:**
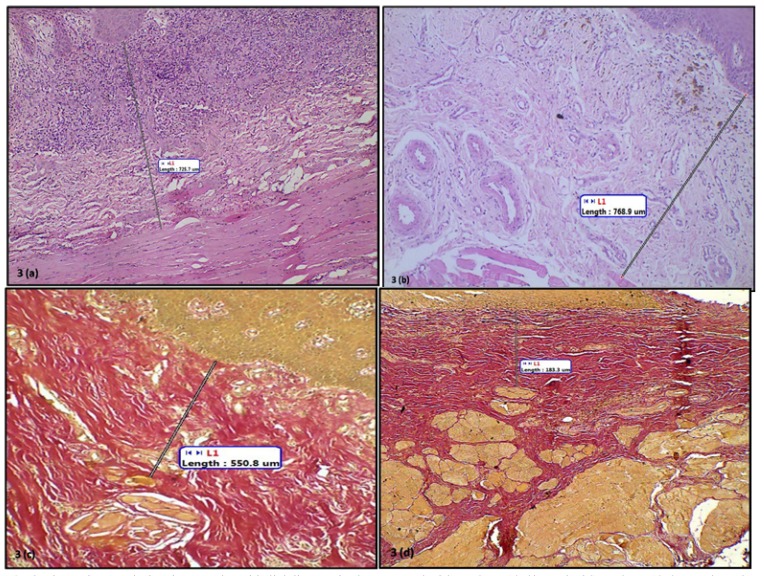
Photomicrograph showing muscle-epithelial distance in. 3a. Very early OSF. H&E,X10. 3b. Early OSF. H&E,X10. 3c. Moderately advanced OSF. Van Geison, X10. 3d. Advanced OSF. Van Geison, X10.

The mean muscle-epithelial distance in control group was 1222.19 ± 19 µm, while in very early, early, moderately advanced and advanced OSF was 732.73 ± 232.81µm, 726.54 ±361.63 µm, 548.36 ± 273.13 and 172.40 ±58.41 µm respectively (Figs. 2,3). A highly significant difference in the muscle-epithelial distance was seen between the control group as compared to early [*P*-value = 0.002], moderately advanced [*P*-value = 0.001] and advanced OSF groups [*P*-value = 0.001]. Even though, a significant difference was found in muscle-epithelium distance when comparing advanced OSF with controls; this data should be interpreted with caution since this group contained only two cases. The difference between muscle epithelial distance in very early OSF as compared to controls was not statistically significant ( Table 2) despite the fact that the distance was decreased. Inter-group comparison however, did not reveal any significant results.

**Table 2:**
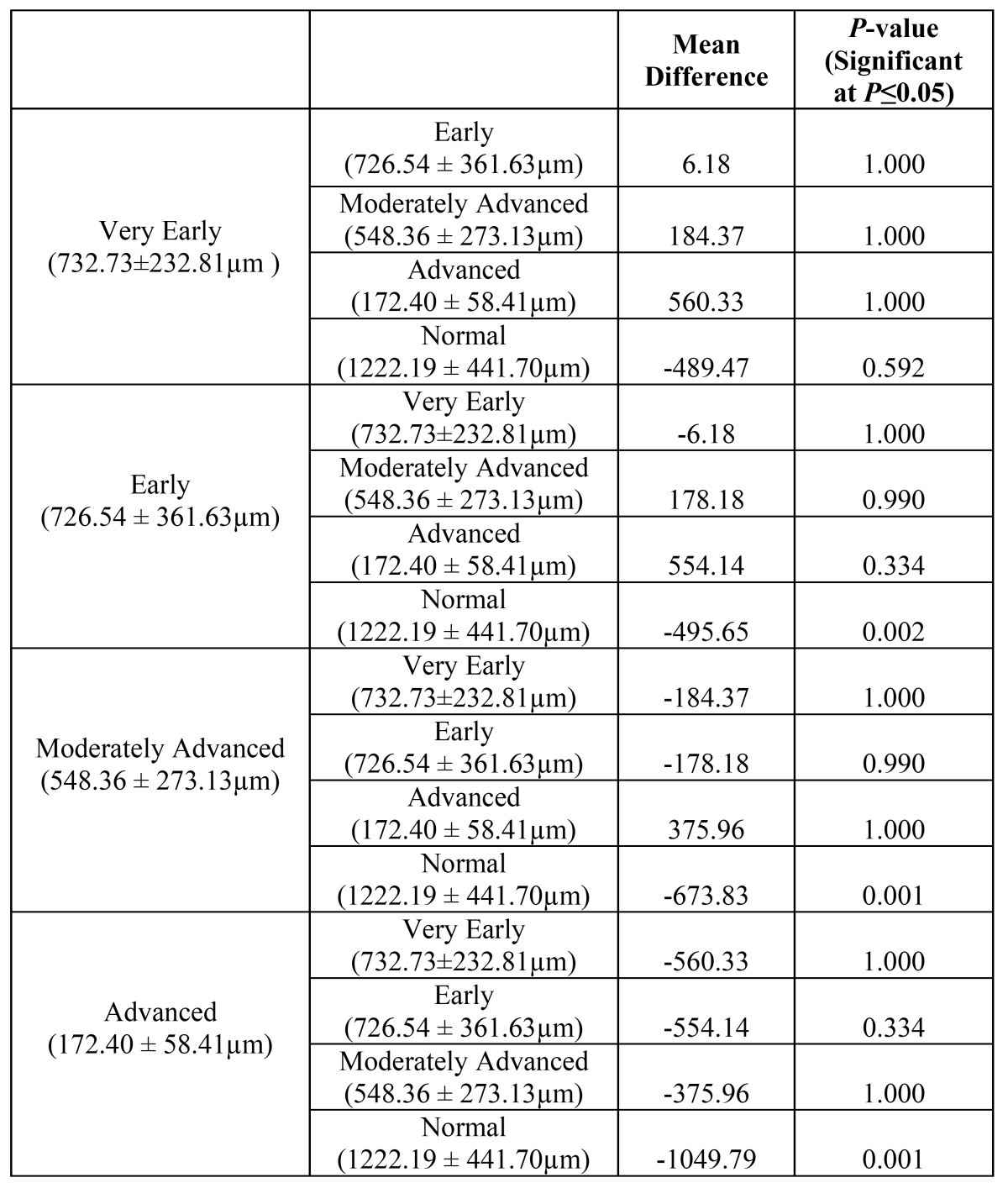
Comparison of muscle- epithelial distance between histopathological groups using post-hoc Bonferroni Test.

No correlation could be discovered between clinical mouth opening and histopathological grade.

**Figure 4:**
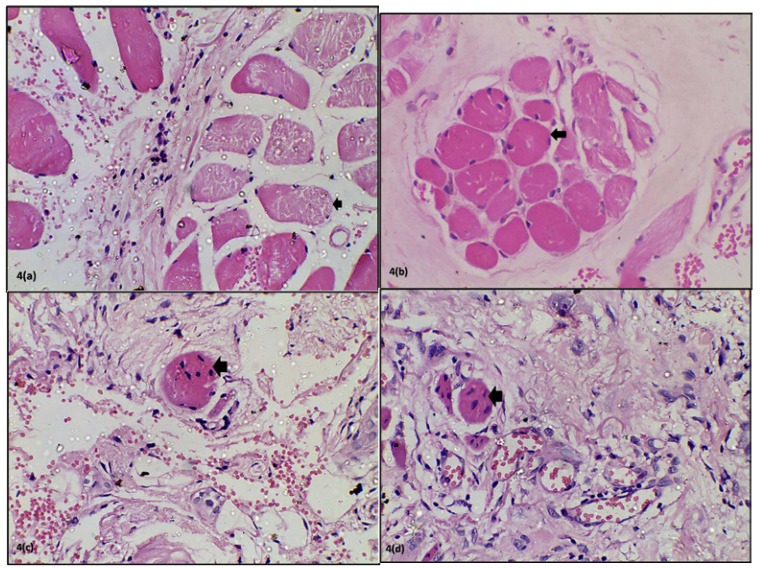
Photomicrograph showing degenerative changes in muscles. 4a. Fragmentation. 4b. Highly eosinophilic areas with loss of striations. 4c. Nucleus internalization. 4d. Multiple pyknotic nuclei.

The results of the degenerative changes observed in muscle have been enlisted in  Table 3. The most commonly observed changes were fragmentation followed by nucleus internalization, highly eosinophilic areas with loss of striations and lastly multiple pyknotic nuclei (Fig. 4). Fragmentation and highly eosinophilic areas with loss of striations were invariably noticed in advanced OSF. The feature of multiple pyknotic nuclei was highly variable with no specificity.

**Table 3:**
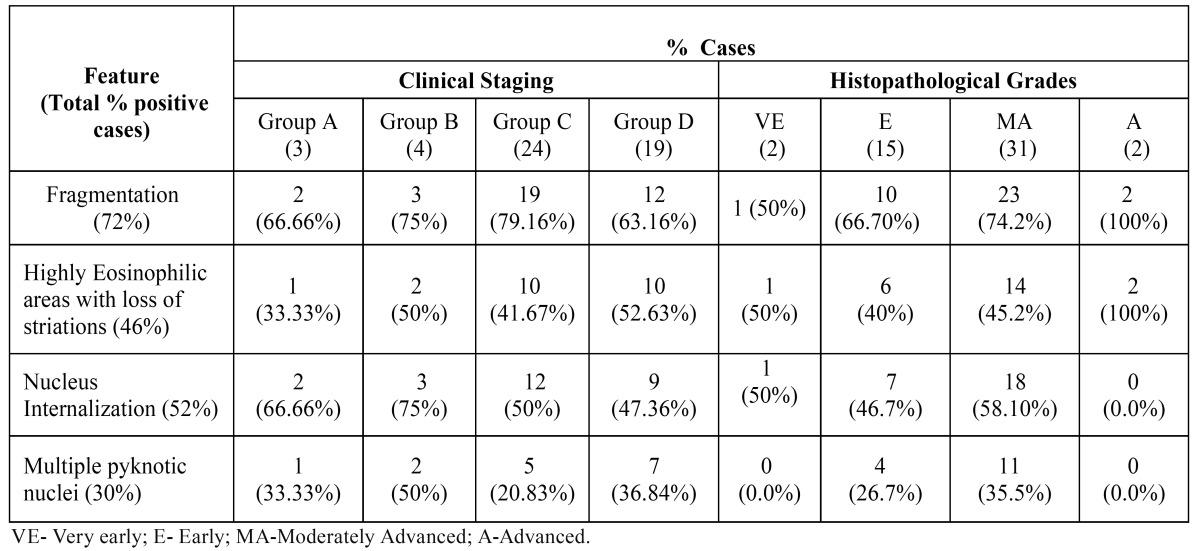
Muscle degenerative changes observed in clinical and histopathological groups of OSF.

## Discussion

OSF is a chronic, insidious disease affecting the oral cavity, pharynx and sometimes esophagus which is characterized clinically by sunken cheeks and progressive inability to open mouth due to loss of elasticity and development of vertical fibrous bands (3).

The anatomical and physiological integrity of the underlying musculature is vital for the degree of mouth opening (10). The present study endeavored to evaluate the histopathological features of muscle-epithelial distance and degenerative changes in the muscles in cases of OSF.

Out of the 50 randomly selected patients of OSF which were included in the present study, majority of the patients had clinical mouth opening less than 29 mm and only 7 patients presented with mouth opening greater than 30 mm. This observation could result from the fact that most patients do not report to a clinician until there is an apparent reduction in the mouth opening accompanied by inability to eat due to vesicle formation and burning sensation. Such symptoms may be ignored by the patient in the early stages of the disease. This discrepancy in the distribution of patients in the different subsets of study groups may have resulted in non-significant correlation comparing mean muscle-epithelial distance and clinical mouth opening while performing inter group comparison. However, a highly significant reduction in muscle-epithelial distance was noted in cases as compared to controls.

Similarly, a highly significant reduction in the muscle-epithelial distance was also noted while comparing the histopathological groups with controls except for very early OSF but even in this group, the mean distance was less than that of controls. Inter group comparison however, did not reveal any significant results.

We hypothesize that this reduction in the muscle-epithelial distance is due to increasing cross-linkages of the collagen present in the stroma lying between the epithelium and the muscle, leading to its condensation. This condensation creates a traction force over the muscle; pulling it towards the epithelium. Electron microscopic studies could provide better insights into such phenomenon.

Binnie and Cawson (4), stated that a characteristic feature of OSF under light microscope was a homogenous, collagenous sub epithelial zone in which degenerating muscle fibres could be seen. Other researchers like El-Labban *et al*. (5), and Sumathi *et al*. (11), have demonstrated ultra structural changes in muscles in OSF. Sumathi *et al*, even stated that restricted mouth opening in OSF depends not only on subepithelial fibrosis but also on the extent of muscle damage. Muscle changes have been observed not only in the buccal mucosa but also in palate and paratubular muscles around Eustachian tube in OSF patients (6). Muscle changes were invariably noticed in more than 90% of the observed histopathological sections in the present study. The features which were most commonly observed has also been included along with photomicrographs.

Fragmentation was seen in 66.66% cases in Group A, 75% cases in Group B, 79% cases in Group C and 63% in Group D. Histopathologically, 100% cases of advanced OSF exhibited fragmentation while 74.19% cases of moderately advanced, 66.7% cases of early OSF and 50% cases of very early OSF showed this feature. El-Labban *et al*. (5) have pointed out that this observation might not hold much significance. These are seen ultra structurally as disrupted and excessively shortened sarcomeres. Such observations have been made in the muscle fibres of patients with normal mouth opening as well as in lesions like oral keratoses and oral lichen planus which are not known to affect muscles. This appearance may result artefactually either as a result of surgical intervention or due to certain fixatives. Contrary to the observations of El-Labban, no muscle changes were found on electron microscopic studies of muscle fibres in OSF patients by Sumathi *et al*. (11).

Some muscle fibres were seen to be stained darkly eosinophilic and the cross striations could not be appreciated in them. This feature varied from 33.3% in Group A to 52.6% in Group D. The ultra structural findings of collection of homogenous material and loss of banding pattern as observed by El-Labban *et al*. (5) and Sumathi *et al*. (11) could be correlated with these light microscopic features. It has been suggested that defects in the plasma membrane lead to calcium entering the sarcoplasm from extra cellular space and acting as a “molecular assassin” (12).

Changes in muscle nuclei are vital signs of pathologic events occurring in the fibre and indicate a progressive degeneration of musculature with advance of the disease process (11). In the present study, nucleus internalization was found in around half of the patients. Nucleus internalization appears to be an early event amongst the degenerative changes as shown by our study. Multiple pyknotic nuclei were found in 60% of this half of patients showing nucleus internalization.

A significant reduction in the muscle-epithelial distance was found between the clinical groups and controls as well as between the histopathological groups and controls. Based on these results we conclude that the reduction in the muscle-epithelial distance may prove to be a significant predictor of OSF progression. To substantiate these results, further studies with larger sample size are needed.

The present study is novel and unique since it is the first of its kind which tries to correlate a clinical parameter (mouth opening) with a new histopathological criterion (distance of muscle fibres from overlying epithelium) in OSF. The study attempts to arrive at a consensus in clinical staging and histological grading of OSF, which has long been a topic of incongruity amongst researchers. Although muscle changes in OSF sections have been described by a very few authors in the past; the detailed description and its validation are still lacking in literature. The present study tries to bring these changes to the forefront; which themselves can be as important as the ongoing process of collagenization of stroma during the pathological advancement of OSF.
